# Treatment Preference Among People With Cystic Fibrosis: The Importance of Reducing Treatment Burden

**DOI:** 10.1016/j.chest.2022.07.008

**Published:** 2022-07-19

**Authors:** Rory A. Cameron, Daniel Office, Jessie Matthews, Mark Rowley, Janice Abbott, Nicholas J. Simmonds, Jennifer A. Whitty, Siobhán B. Carr

**Affiliations:** a Norwich Medical School, University of East Anglia, Norwich, England; b National Institute for Health Research, Applied Research Collaboration, East of England, Cambridge, England; c Adult Cystic Fibrosis Centre, Royal Brompton Hospital, London, England; d patient representative, York, England; e School of Psychology, University of Central Lancashire, Preston, England; f National Heart and Lung Institute, Imperial College, London, England; g Department of Paediatric Respiratory Medicine, Royal Brompton Hospital, London, England; h Evidera, London, England

**Keywords:** CFTR modulators, cystic fibrosis, discrete choice experiment, patient and public involvement, patient preference, treatment burden, CF, cystic fibrosis, CFQ-R, Cystic Fibrosis Questionnaire-Revised, CFRD, cystic fibrosis-related diabetes, CFTR, cystic fibrosis transmembrane conductance regulator, DCE, discrete choice experiment, ETI, elexacaftor-tezacaftor-ivacaftor, GERD, gastroesophageal reflux disease, HRQoL, health-related quality of life, LC, latent class, MNL, multinomial logit, PERT, pancreatic enzyme replacement therapy, ppFEV_1_, FEV_1_ % predicted, PwCF, people with cystic fibrosis

## Abstract

**Background:**

There is a growing consensus that the perspective of the patient should be considered in the evaluation of novel interventions.

**Research Question:**

What treatment outcomes matter to people with cystic fibrosis (CF), and what trade-offs would they make to realize these outcomes?

**Study Design and Methods:**

Adults attending a specialist CF center were invited to complete an online discrete choice experiment (DCE). The DCE required participants to evaluate hypothetical CF treatment profiles, defined by impact on lung function, pulmonary exacerbations, abdominal symptoms, life expectancy, quality of life, inhaled medicine usage, and physiotherapy requirement. Choice data were analyzed, using multinomial logit and latent class models.

**Results:**

One hundred and three people with CF completed the survey (median age, 35 years; range, 18-76 years); 52% were female; mean FEV_1_ % predicted, 69% [SD, 22%]). On average, an improvement in life expectancy by 10 years or more had the greatest impact on treatment preference, followed by a 15% increase in lung function. However, it was shown that people would trade substantial reductions in these key outcomes to reduce treatment time or burden. Preference profiles were not uniform across the sample: three distinct subgroups were identified, each placing markedly different importance on the relative importance of both life expectancy and lung function compared with other attributes.

**Interpretation:**

The relative importance of treatment burden to people with CF, compared with life expectancy and lung function, suggests it should be routinely captured in clinical trials as an important secondary outcome measure. When considering the patient perspective, it is important that decision-makers recognize that the values of people with CF are not homogeneous.

**Take-home Points**

**Study Question:** What treatment outcomes matter to people with cystic fibrosis, and what trade-offs would they make to realize these outcomes?

**Results:** Improving life expectancy was found to be the most important outcome in this study, but people with cystic fibrosis were prepared to accept substantial reductions in this outcome, and in lung function to reduce their treatment burden.

**Interpretation:** Awareness of the priorities of people with cystic fibrosis with regards to their treatment outcomes may improve decision making both at the policy and at the clinic levels.

FOR EDITORIAL COMMENT, SEE PAGE 1225

Cystic fibrosis (CF) is a rare genetic condition with an estimated live-birth incidence of between 1 in 2,000 and 1 in 6,000 in populations of European and Middle Eastern descent.^1^ Most people with CF (PwCF) will require lifelong treatment involving frequent hospital visits and admissions and rigorous daily therapy regimens.

The average daily time associated with treatment has been estimated at over 1.5 h.^2^ This high level of treatment burden has a substantial impact on health-related quality of life (HRQoL), and is associated with reduced adherence.^3^^,^^4^ PwCF have been shown to rationalize which treatments they take, depending on how they fit into their daily-life commitments,^2^ with the lowest levels of adherence for treatments perceived to be more burdensome.^5^^,^^6^ Low adherence commonly equates to poorer outcomes,^7^^,^^8^ and has been associated with elevated costs for acute medical care,^9^ and ultimately wasted medical resources.^10^

Recent surveys of CF communities have identified simplification of treatment burden as a key research priority.^11^^,^^12^ The recent introduction of cystic fibrosis transmembrane conductance regulator (CFTR) modulator therapies is transforming the outcomes and prognosis for many PwCF, with evidence emerging that their introduction is associated with reduced use of other treatments.^13^ To date, however, these innovations have been designed to be additive to existing regimens, and therefore reduction of burden of treatment remains a priority.

Understanding how patients perceive and prioritize potentially competing outcomes is becoming increasingly important to the development and delivery of new CF therapies and regimens. A criticism of evaluations of new CF therapies is that current assessments of a drug’s value either disregard or are insensitive to patient preferences, or the benefits they prioritize.^14^ As the management of CF evolves, lower treatment burden is anticipated to become a cornerstone of the value that new CF therapies can bring to patients.^2^^,^^7^ At present, there is no agreed approach for technology assessments to objectively consider the values and priorities of patients,^15^ although there is a growing consensus that such assessments should, in some systematic way, incorporate the patient perspective.^16^^,^^17^

This study sought to understand treatment preferences from the perspective of PwCF, the impact of treatment outcome on choice of treatment, and to quantify the trade-offs that people were willing to make between these outcomes. We focus on key clinical outcomes (lung function, life expectancy, and HRQoL) and known drivers of significant treatment burden (physiotherapy, inhaled medicines, pulmonary exacerbation, and pancreatic enzyme replacement therapy [PERT]). The primary objective of this research was to develop a set of metrics (marginal effects) that indicate the relative importance of different treatment outcomes for PwCF. We also include an exploratory analysis of how these preferences vary across the CF population.

## Study Design and Methods

This research formed part of VALU-CF (Evidence-Based Valuation of Patient Outcomes in Cystic Fibrosis), a cross-sectional study focused on the measurement and valuation of CF-specific HRQoL.^18^ It uses a discrete choice experiment (DCE) to characterize the treatment preferences of PwCF. The DCE is a choice-based approach to eliciting preferences.^19^ The approach enables researchers to estimate the relative importance of the characteristics, or “attributes,” of an intervention (eg, dosing regimen, and efficacy of a drug). Each attribute may have a number of different “levels” (dosing regimen, eg, might be daily or twice daily). Examination of the relative preference for different levels within each attribute facilitates estimation of the trade-offs that individuals are willing to accept between attributes. The theoretical basis underpinning DCEs is an assumption that individuals value interventions based on their component attributes,^20^ and the likelihood of choosing one intervention over another is a function of the attributes of each intervention.^19^^,^^21^ The attributes investigated in this study include the treatment outcome and burden impacts of a hypothetical new oral drug for CF.

### Development of the DCE Survey

Development of the survey instrument followed good research practice guidelines for DCEs, and survey design.^22^^,^^23^ The VALU-CF study and the DCE survey were approved by the National Health Service Health Research Authority (REC 19/YH/0423), and all participants provided informed consent.

**Figure 1:**
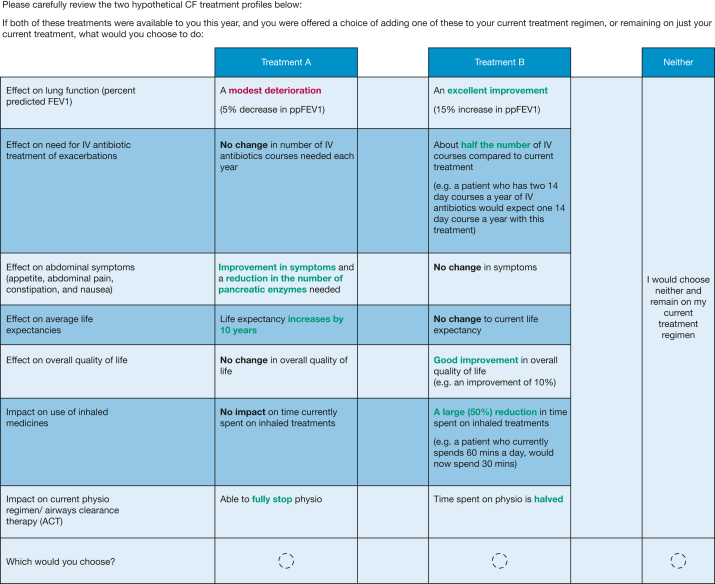
An example choice scenario from the discrete choice experiment survey. CF = cystic fibrosis; ppFEV1 = FEV_1_ % predicted

The DCE presented each participant with 12 choice scenarios in which they were asked to choose between different hypothetical treatment options. An example choice scenario is shown in Figure 1.

The treatment options were defined by seven attributes (Table 1). All treatment options were described as once-daily tablets with a very low risk of serious adverse events. The participant was asked to make a choice of adding one of the two treatments to their daily regimen, or opting out of the additional drug therapy. The 12 tasks were presented to each participant in a randomized order. The combination of treatment profiles presented in the choice scenarios were generated using Ngene software (ChoiceMetrics), employing a D-efficient design (further experimental design details are presented in e-Appendix 1).

**Table 1:** Attributes and Levels

Attribute	Level
Effect on lung function (ppFEV_1_)	No change A modest deterioration (5% decrease in ppFEV_1_) A modest improvement (5% increase in ppFEV_1_) An excellent improvement (15% increase in ppFEV_1_)
Effect on need for IV antibiotic treatment of exacerbations	No change in number of IV antibiotic courses needed each year About one-half the number of IV courses compared with current treatment
Effect on abdominal symptoms (appetite, abdominal pain, constipation, and nausea)	No change in symptoms Improvement in symptoms Improvement in symptoms and a reduction in the number of pancreatic enzymes needed
Effect on average life expectancy	No change to current life expectancy Life expectancy increases by 5 y Life expectancy increases by 10 y Life expectancy increases by 15 y
Effect on overall quality of life	No change in overall quality of life Good improvement in overall quality of life (eg, an improvement of 10%) Excellent improvement in overall quality of life (eg, an improvement of 20%)
Impact on use of inhaled medicines	No impact on time currently spent on inhaled treatments A modest (25%) reduction in time spent on inhaled treatments A large (50%) reduction in time spent on inhaled treatments
Impact on current physiotherapy regimen/airway clearance therapy	No impact on the time currently spent on physiotherapy Time spent on physiotherapy is halved Able to fully stop physiotherapy

ppFEV_1_ = FEV_1_ % predicted.

Key treatment outcome attributes to be considered were first identified by expert opinion, and through the literature. The attribute list was refined and finalized based on feedback from a focus group with PwCF, and carers of PwCF, conducted in June 2019. The design included seven attributes that considered the impact of a hypothetical new treatment on: lung function (change in FEV_1_ % predicted [ppFEV_1_]), life expectancy, frequency of pulmonary exacerbations (change in number of days on IV antibiotics), GI symptoms and the need for PERT, overall quality of life, time spent on inhaled medicines, and time spent on physiotherapy. The levels of the attributes (informed by evidence, consultation with clinical and outcomes experts, and PwCF) were chosen to represent feasible and clinically meaningful outcomes of hypothetical new treatments analogous to triple-combination CFTR modulator therapy.^24^

The survey also contained predominantly closed questions on demographics, HRQoL (EQ-5D-5L and self-rated health visual analog scale [EuroQol Group]), treatment complexity (e-Appendix 2, e-Table 1),^25^ and treatment burden, reported elsewhere.^26^ It was estimated that survey completion would take 20 to 25 min.

### Recruitment and Data Collection

Administered online and hosted by SurveyEngine, the survey ran between July and October 2020. Adopting a purposive sampling approach, 276 adults with CF attending the Royal Brompton Hospital Adult CF Centre were emailed an invitation to participate. Inclusion criteria required the participants to have a CF diagnosis, be over the age of 18 years, and have the mental capacity to complete the survey. Inpatients experiencing acute exacerbations and judged by the research nurse to be too unwell to be approached were excluded. A £10 online voucher was offered as an incentive to participate, and respondents were sent up to two reminder emails. The survey was preceded by an electronic participant information sheet and an informed consent section that included optional linkage of survey data to the participant’s UK CF Registry data.

The survey was paused after 13 completions in an initial “pilot” phase. On the basis of feedback, minor formatting changes were made to the display of the attributes to highlight the elements of the attributes that changed across choice sets, and the direction of that change.

### Statistical Analysis

All analyses were conducted with Stata IC (StataCorp). Incomplete surveys were omitted from the analysis.

The DCE data were analyzed initially with a multinomial logit (MNL) model. The model included a constant to represent the choice of declining either treatment in each choice scenario. In the base case model (MNL model 1), all attribute variables were dummy coded; however, to simplify trade-off calculations a model (MNL model 2) with the lung function and life expectancy attributes coded as continuous variables was also estimated.

Trade-offs were estimated against both lung function and life expectancy by calculating the ratios of their marginal effects to those of other attributes to provide the marginal rate of substitution per unit change in ppFEV_1_ or life expectancy. Trade-off CIs were estimated, using the delta method.^27^

A simplifying assumption of the MNL model is that preferences are uniform across the sample. To address this limitation, a latent class (LC) model was estimated with lclogit2, a user-written Stata program.^28^ LC models extend the MNL by incorporating unobserved heterogeneity of preferences across participants. The LC model assumes a discrete number of classes of preference profile within the population, whose membership is characterized by unobserved variables.^28^^,^^29^ Final model specification was guided by minimization of the Bayesian information criterion. Probability of class membership was estimated for each participant and used to designate specific classes.

Methods for scoring, and detailed analysis of the treatment burden measures in this study (including their relative performance), have been published separately.^26^ The cross-walk algorithm was used to score the EQ-5D-5L measure.^30^

## Results

### Survey Population

The survey was completed by 103 PwCF, giving a response rate of 37%. All participants consented for their registry data to be linked with the survey; however, an error in participant tracking meant that we were unable to identify and link the data of two participants. The choice data for these two participants were retained in the DCE modeling. Five patients were excluded from recruitment, (three for mental health reasons, two because they were new to the service); no patients were excluded because of severity of CF.

The survey sample participants (Table 2), 52% female with a median age of 35 years (range, 18-76 years), and a ppFEV_1_ of 69% (SD, 22), showed no differences from the center’s CF population regarding lung function, BMI, or use of mucolytics or osmotic therapies (e-Table 2).^31^ The sample was also broadly representative of the UK adult CF population against key clinical and treatment characteristics.^31^ Mean scores for HRQoL (EQ-5D-5L index, 0.77) and treatment burden (Cystic Fibrosis Questionnaire-Revised [CFQ-R] treatment burden domain, 54) were similar to those for patients with mild disease in a study by Acaster et al*.*^32^ On average, sample participants spent 92 min managing a total of 14 treatments each day.

**Table 2:** Characteristics of Survey Participants

Characteristic	No. in Sample	Mean (SD) or No. (%)	Median (IQR)
Demographics			
Age, y	101	36 (11)	35 (17)
Sex, female	101	52 (52%)	…
Clinical measures			
BMI	101	23 (3.2)	23 (3.6)
ppFEV_1_	101	69 (22)	69 (30)
Mild (> 70%)^a^	101	50 (50%)	…
Moderate (40%-70%)	…	39 (39%)	…
Severe (< 40%)	…	12 (12%)	…
absFEV_1_, L	99	2.5 (1.1)	2.3 (1.4)
ppFVC	94	85 (20)	87 (27)
Absolute FVC, L	94	3.7 (1.1)	3.6 (1.5)
Diagnosis of GERD	101	42 (42%)	…
Diagnosis of CFRD	101	29 (29%)	…
Treatment characteristics			…
Treatment complexity score	101	22 (7.4)	23 (9)
Total treatment time, min/d	103	92 (71)	85 (65)
Physiotherapy time, min/d	103	38 (33)	30 (40)
Inhaled medicines time, min/d	103	43 (38)	30 (40)
No. chronic treatments	101	13 (4.8)	13 (5)
Prescribed CFTR modulator	101	65 (65%)	…
Ivacaftor	101	4 (4%)	…
Tezacaftor/ivacaftor	…	29 (29%)	…
Elexacaftor/tezacaftor/ivacaftor	…	33 (33%)	…
Received IV antibiotics in last year	101	36 (36%)	
No. of IV antibiotic courses in last year^b^	36	2.6 (2.1)	2 (3)
HRQoL and treatment burden measures			
EQ-5D Index score	103	0.77 (0.19)	0.77 (0.2)
EQ-5D VAS score	103	75 (16)	80 (22)
CFQ-R treatment burden domain score	103	54 (23)	56 (33)
CFQoL treatment burden domain score	103	64 (26)	67 (40)

absFEV_1_ = absolute FEV_1_; CFRD = cystic fibrosis-related diabetes; CFTR = cystic fibrosis transmembrane conductance regulator; CFQoL = cystic fibrosis-related quality of life; CFQ-R = Cystic Fibrosis Questionnaire-Revised; EQ-5D = EuroQol-5 Dimension; GERD = gastroesophageal reflux disease; HRQoL = health-related quality of life; IQR = interquartile range; ppFEV_1_ = FEV_1_ % predicted; VAS = visual analog scale.

a Percentages do not sum to 100, due to rounding.

b For those who received at least one IV antibiotic course.

### Outcome Preferences

The 103 participants, each with 12 choice scenarios, generated 1,236 observations for analysis. All responses were included in the analysis.^33^ Participants chose not to take up either treatment for a total of 73 (6%) of the choice scenarios, with 1 participant (1%) opting out of treatment in all 12 choice scenarios. One participant selected option B for all scenarios, which might suggest task nonattendance.

### Multinomial Logit Model Results

**Figure 2:**
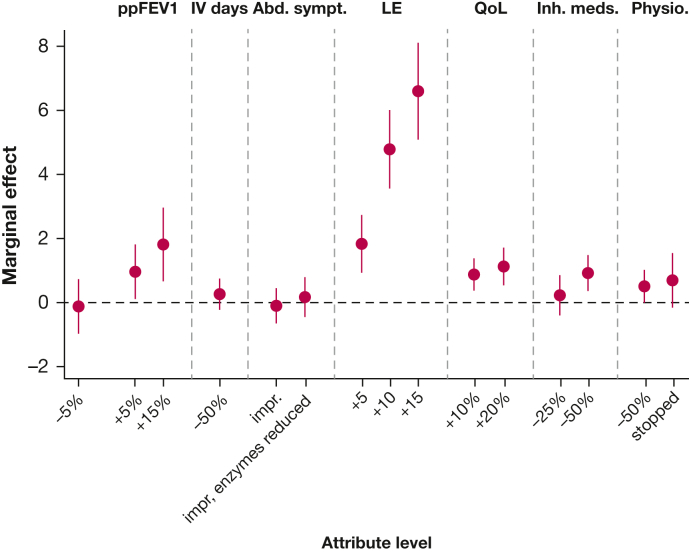
MNL model 1 results. Values represent mean marginal effect, ± 95% CI. The marginal effect for no change (reference level) is represented by the dashed horizontal line. Abd. sympt. = abdominal symptoms; impr = symptoms improved; impr, enzymes reduced = symptoms reduced and pancreatic enzymes reduced; Inh. meds. = inhaled medications; LE = life expectancy (years); MNL = multinomial logit; Physio. = physiotherapy; ppFEV1 = FEV_1_ % predicted; QoL = overall quality of life.

The MNL model estimates coefficients that may be interpreted as mean marginal effects for treatment outcomes. These results (MNL model 1) are presented in Table 3 and Figure 2.

**Table 3:** Multinomial Logit Model 1 Results

Attribute	Parameter (Level)	Marginal Effect **(**95% CI**)**
	Opt-out constant	0.4 (–0.35 to 1.16)
Lung function	Modest deterioration (–5%)	–0.45^a^ (–0.79 to –0.11)
	No change	Referent
	Modest improvement (+5%)	0.65^b^ (0.36 to 0.95)
	Excellent improvement (+15%)	1.24^b^ (0.77 to 1.72)
Need for IV antibiotics	No change	Referent
	One-half the number of IV courses	0.27^a^ (0.1 to 0.44)
Abdominal symptoms	No change	Referent
	Improvement in symptoms	0.26^a^ (0.02 to 0.51)
	Improvement in symptoms and a reduction in pancreatic enzymes	0.37^b^ (0.17 to 0.58)
Life expectancy	No change	Referent
	Increases by 5 y	0.55^b^ (0.27 to 0.82)
	Increases by 10 y	1.85^b^ (1.45 to 2.26)
	Increases by 15 y	2.34^b^ (1.83 to 2.85)
Overall quality of life	No change	Referent
	Good improvement (+10%)	0.31^a^ (0.12 to 0.5)
	Excellent improvement (+20%)	0.65^b^ (0.43 to 0.88)
Use of inhaled medicines	No change	Referent
	A modest reduction in time spent (–25%)	0.18^a^ (0.01 to 0.35)
	A large reduction in time spent (–50%)	0.3^a^ (0.11 to 0.48)
Physiotherapy/ACT	No change	Referent
	Time spent on physiotherapy is halved	0.19^a^ (0.07 to 0.32)
	Able to fully stop physiotherapy	0.51^b^ (0.26 to 0.75)
Model statistics	No. of observations, 1,236; McFadden *R*^2^, 0.32; LL, –924; AIC, 1,882; BIC, 1,988	

ACT = airway clearance therapy; AIC = Akaike information criteria; BIC = Bayesian information criterion; LL = log likelihood.

a *P* < .05.

b *P* < .001.

The nonsignificant opt-out coefficient indicated no propensity for participants to opt out of treatment. Marginal effects for all attributes were significantly different from zero and positive (with the exception of a 5% reduction in ppFEV_1_, which was negative, as expected) and increased in magnitude in a logically consistent manner. People showed a preference for improvements in life expectancy of 10 years or more over all other attributes. Improvement in lung function also had a notable impact on choice of treatment. When considering treatment burden-related attributes, the greatest preference was shown for stopping physiotherapy, followed by reduced PERT, coupled with improved abdominal symptoms.

Table 4 summarizes willingness to accept a reduction in lung function or additional life expectancy for an improvement in other outcomes (based on MNL model 2; e-Table 3). The largest trade-offs were for an “excellent improvement in quality of life,” with people prepared to accept a reduction in ppFEV_1_ of 8.2% (95% CI, 5.8-10.7), or 4.2 years of additional life expectancy (95% CI, 3.1-5.4) on average. People were also prepared to accept notable reductions in lung function or additional life expectancy to reduce their treatment burden: 6.1 ppFEV_1_ (95% CI, 3.6-8.7) or 3.2 years of additional life expectancy (1.8-4.5) to fully stop physiotherapy; 5.3 ppFEV_1_ (3.3-7.3) or 2.7 years of additional life expectancy (1.6-3.8) if abdominal symptoms improved with a concomitant reduction in PERT; and 4.4 ppFEV_1_ (2.6-6.3) or 2.3 years of additional life expectancy (1.3-3.3) to halve the time spent on inhaled medicines.

**Table 4:** Willingness to Accept a Reduction in ppFEV_1_ or Additional Life Expectancy Against Other Treatment Outcomes

Attribute	Acceptable Reduction in ppFEV_1_ (95% CI)	Acceptable Reduction **in** Additional Life Expectancy^a^ (95% CI)
Excellent improvement (+20%) in QoL	8.2 (5.8-10.7)	4.2 (3.1-5.4)
Able to fully stop physiotherapy	6.1 (3.6-8.7)	3.2 (1.8-4.5)
Abdominal symptoms improved and enzymes reduced	5.3 (3.3-7.3)	2.7 (1.6-3.8)
A large reduction in time spent (–50%) on inhaled medicines	4.4 (2.6-6.3)	2.3 (1.3-3.3)
Abdominal symptoms improved	4.2 (1.7-6.8)	2.2 (0.8-3.5)
Good improvement (+10%) in QoL	3.5 (1.2-5.8)	1.8 (0.7-2.9)
Time spent on physiotherapy is halved	2.7 (1.2-4.3)	1.4 (0.6-2.2)
IV days halved	2.4 (0.7-4.1)	1.2 (0.3-2.2)
Per**-**year increase in life expectancy	1.9 (1.5-2.4)	…
A modest reduction in time spent (–25%) on inhaled medicines	1.9 (0.0-3.8)	1.0 (0.0-2.0)
Per 1% increase in predicted FEV_1_	…	0.5 (0.4-0.6)

ppFEV_1_ = FEV_1_ % predicted; QoL = quality of life.

a Additional life expectancy should be interpreted as the additional life expectancy conferred by the hypothetical treatments presented in the discrete choice experiment, beyond existing life expectancy.

In secondary analyses, we investigated the impact on preferences of having a CFTR modulator prescription (e-Fig 1, e-Table 4), and of responding to the survey after it was announced that elexacaftor-tezacaftor-ivacaftor (ETI) would be reimbursed in the United Kingdom (e-Fig 2). No significant differences in preferences were found for those who completed the survey before ETI reimbursement compared with those who completed the survey after its general availability. Those not prescribed CFTR modulators tended to be less concerned about modest reductions in lung function, and to value more highly improvements in abdominal symptoms as well as significant quality-of-life improvements. As a consequence, this group was prepared to accept larger reductions in lung function to improve their abdominal symptoms. Those not prescribed CFTR modulators tended to have a lower treatment burden than those taking modulators, but no other significant differences in clinical or demographic characteristics were observed (e-Table 5).

**Table 5:** Latent Class Model Results

Attribute	Parameter (Level)	Class 1^a^ (Predicted Share, 43%^b^)		Class 2 (Predicted Share, 47%)		Class 3 (Predicted Share, 10%)	
		Marginal Effect	95% CI	Marginal Effect	95% CI	Marginal Effect	95% CI
	Opt-out constant	0.58	–0.81 to 1.98	–0.72	–1.7 to 0.25	4.30^c^	2.16 to 6.44
Lung function	Modest deterioration (–5%)	–0.11	–0.98 to 0.75	–1.11^c^	–1.64 to –0.57	–0.92	–1.91 to 0.06
	No change	Referent					
	Modest improvement (+5%)	0.97^d^	0.11 to 1.83	0.97^c^	0.6 to 1.35	0.2	–0.78 to 1.19
	Excellent improvement (+15%)	1.85^e^	0.7 to 3	1.73^c^	1.16 to 2.31	0.83	–0.35 to 2.01
Need for IV antibiotics	No change	Referent					
	One-half the number of IV courses	0.27	–0.24 to 0.78	0.52^c^	0.27 to 0.77	–0.21	–0.83 to 0.42
Abdominal symptoms	No change	Referent					
	Improvement in symptoms	–0.09	–0.68 to 0.5	0.57^e^	0.23 to 0.91	0.17	–0.58 to 0.93
	Improvement in symptoms and a reduction in pancreatic enzymes	0.21	–0.35 to 0.77	0.67^c^	0.32 to 1.02	0.34	–0.47 to 1.15
Life expectancy	No change	Referent					
	Increases by 5 y	1.85^c^	0.94 to 2.75	0.49	–0.02 to 1.01	1.79^e^	0.45 to 3.12
	Increases by 10 y	4.79^c^	3.57 to 6.02	1.49^c^	0.96 to 2.01	2.45^e^	1.04 to 3.86
	Increases by 15 y	6.62^c^	5.08 to 8.16	1.71^c^	1.1 to 2.32	3.05^c^	1.51 to 4.59
Overall quality of life	No change	Referent					
	Good improvement (+10%)	0.89^c^	0.37 to 1.42	0.29	0 to 0.58	0.46	–0.35 to 1.27
	Excellent improvement (20%)	1.14^c^	0.57 to 1.71	0.9^c^	0.56 to 1.25	0.72	–0.08 to 1.52
Use of inhaled medicines	No change	Referent					
	A modest reduction in time spent (25%)	0.24	–0.37 to 0.85	0.20	–0.11 to 0.5	1.13^e^	0.29 to 1.97
	A large reduction in time spent (50%)	0.95^e^	0.41 to 1.49	0.34^e^	0.03 to 0.65	0.99^e^	0.08 to 1.9
Physiotherapy/ACT	No change	Referent					
	Time spent on physiotherapy is halved	0.53^e^	0.02 to 1.04	0.17	–0.1 to 0.45	0.45	–0.32 to 1.22
	Able to fully stop physiotherapy	0.71	–0.17 to 1.59	0.80^c^	0.47 to 1.13	0.91^e^	0.08 to 1.74
Model statistics	No. of observations, 3,708; McFadden *R*^2^, N/A; LL, –697; AIC, 1,501; BIC, 1,831						

ACT = airway clearance therapy; AIC = Akaike information criterion; BIC = Bayesian information criterion; LL = log likelihood; N/A = not applicable.

a As marginal effects are calculated relative to the reference level for each class, they are not directly comparable across classes; the ratios between effects, however, may be directly compared.

b Probability of class membership equates to percentage class share of the population.

c *P* < .001.

d *P* < .1.

e *P* < .05.

### Latent Class Model Results

A model with three latent classes was deemed to be both the best fit and the most logically coherent model. On the basis of probability of class membership, the model predicted that 43% of the sample fell into class 1, 47% in class 2, and 10% in class 3.

**Figure 3:**
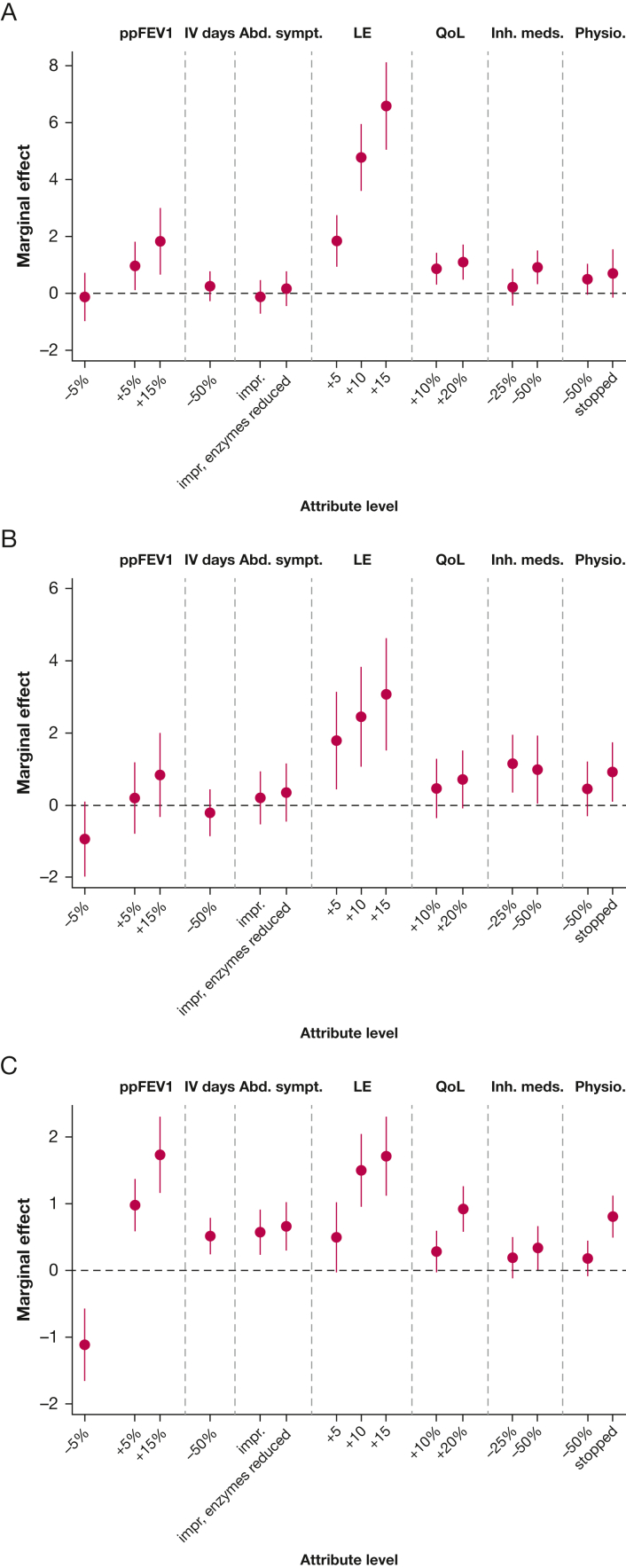
A-C, Latent class model results for (A) class 1, (B) class 2, and (C) class 3. Values represent mean marginal effect, ± 95% CI. The marginal effect for no change (reference level) is represented by the dashed horizontal line. Abd. sympt. = abdominal symptoms; impr = symptoms improved; impr, enzymes reduced = symptoms reduced and pancreatic enzymes reduced; Inh. meds. = inhaled medications; LE = life expectancy (years); Physio. = physiotherapy; ppFEV1 = FEV_1_ % predicted; QoL = overall quality of life.

The results of the latent class model are shown in Table 5 and Figure 3. Consistent with the MNL model, improvements in life expectancy were overall the strongest drivers of preference; however, the strength of this preference relative to other attributes differs markedly across the classes. Class 1 is primarily characterized by improvements in life expectancy. They were indifferent to a modest reduction in lung function, and to reductions in most treatment burden-related and abdominal symptom outcomes. However, a 50% reduction in time spent on inhaled medicines was viewed as equivalent to a modest (5%) improvement in lung function or a 20% improvement in quality of life. Conversely, class 2 strongly valued an increase in lung function and was inclined to avoid a decrease in lung function, and reduced treatment burden, with stopping physiotherapy the preferred treatment burden outcome. Owing to the small sample membership for class 3, preferences should be interpreted with caution; however, this class had a stronger likelihood of opting out of an additional treatment and appeared indifferent to changes in lung function.

Characteristics of the predicted classes are summarized in Table 6. Although some significant predictors of class membership were observed, few of the a priori-specified participant characteristics were found to be strong predictors of class membership. There were statistically significant differences in lung function between participants who were likely to belong to class 1 and class 2 (absolute FEV_1_, *P* = .03; absolute FVC, *P* = .01). Nominally, class 2 had an increased likelihood of a CFRD diagnosis, longer overall treatment time, and lower HRQoL than class 1. There were no differences between class 1 and class 2 in terms of age, sex, or treatment burden or complexity scores. Compared with class 1, class 3 participants were more likely to be female (*P* = .06), had a lower treatment complexity score (*P* = .01), and lower treatment burden as measured by the CFQ-R (*P* = .03). Nominally, class 3 spent less time on all forms of treatment, were less likely to receive IV antibiotics, and had a superior HRQoL compared with both classes 1 and 2.

**Table 6:** Comparison of Predicted Class Member Characteristics

Characteristic	Class 1 (n = 45)	Class 2 (n = 48)	Class 3 (n = 10)	*P* Value^a^
Demographics				
Age, y	36	36	36	.99
Sex, % female	43	54	78^b^	.1
Clinical measures				
ppFEV_1_	71	64	85^b^	.02
Mild, %	52	43	70	.4
Moderate, %	36	43	30	
Severe, %	11	15	0	
absFEV_1_, L	2.72	2.22^c^	2.51	.08
ppFVC	88	80^b^	99	.01
absFVC, L	3.94	3.35^c^	3.88	.04
Diagnosis of GERD, %	41	42	44	.98
Diagnosis of CFRD, %	25	33	22	.6
BMI	23	23	24	.7
Treatment characteristics				
Treatment complexity score	22	23	16^c^	.01
Total treatment time, min/d	91	104	42^c^	.04
Physiotherapy time, min/d	38	42	17^b^	.09
Inhaled medicines time, min/d	45	46	19^b^	.1
No. chronic treatments	13	14	9^c^	.02
Prescribed CFTR modulator, %	70	60	67	.6
Prescribed elexacaftor/tezacaftor/ivacaftor, %	34	29	44	.7
Received IV antibiotics in last year, %	39	35	22	.6
Number of IV antibiotic courses in last year^d^	2.5	2.9	1.5	.6
HRQoL and treatment burden measures				
EQ-5D Index score	0.80	0.72^c^	0.88^b^	.02
EQ-5D VAS score	75	74	80	.6
CFQ-R treatment burden domain score	54	50	74^c^	.007
CFQoL treatment burden domain score	63	63	76	.3
MTBQ index score (reversed)	80	81	90^c^	.09

absFEV_1_ = absolute FEV_1_; absFVC = absolute FVC; CFQ-R = Cystic Fibrosis Questionnaire-Revised; CFQoL = cystic fibrosis-related quality of life; CFRD = cystic fibrosis-related diabetes; CFTR = cystic fibrosis transmembrane conductance regulator; EQ-5D = EuroQol-5 Dimension; GERD = gastroesophageal reflux disease; HRQoL = health-related quality of life; MTBQ = Multimorbidity Treatment Burden Questionnaire; ppFEV_1_ = FEV_1_ % predicted; ppFVC = FVC % predicted.

a One-way analysis of variance tests used for continuous variables, Pearson’s χ^2^ used for categorical variables.

b *P* < .1, *t*-test comparisons against class 1.

c *P* < .05, *t*-test comparisons against class 1.

d For those who received at least one IV antibiotic course.

## Discussion

In 2018 a survey of PwCF identified treatment burden as their number one priority research topic.^11^ The improved prognosis that many PwCF can expect as a consequence of more effective therapies is likely to reinforce this priority. To our knowledge, this is the first study to explicitly quantify the relative importance to PwCF of reducing diverse aspects of treatment burden related to the management of CF.

As would be expected for a life-limiting chronic respiratory condition, PwCF placed greatest importance on treatments that will extend life expectancy; improvements to lung function are also very important. However, the extent to which people are willing to trade gains in these two major outcomes to reduce their treatment burden or, for example, reduce their abdominal symptoms underscores the relative importance to the patient of these aspects of their disease and its management. Of the treatment burden-related outcomes, people were prepared to accept the largest reductions to stop physiotherapy, followed by reducing PERT, coupled with an improvement in GI symptoms, and halving of inhaled medicines. The findings are in broad agreement with a study suggesting that airway clearance therapy, nebulized antibiotics, and PERT are the top three most burdensome CF treatments.^34^ The trade-offs reported may be additive (assuming no interactions or dependencies between attributes), suggesting, for example, that people may be willing to accept a reduction in lung function of more than 5% predicted FEV_1_ for a new treatment that conferred a 50% reduction in both IV days and time spent on physiotherapy. An investigation of the impact of CFTR modulator prescription on preferences suggests that those not prescribed modulators place greater importance on reducing abdominal symptoms than those who are. This finding aligns with emerging evidence that the modulators improve digestive outcomes in CF,^35^ and may suggest that after initiation on a CFTR modulator, these symptoms become less of a priority for the patient.

A secondary objective of the research was to explore how treatment preferences vary across the CF population; in the latent class model, we identified three distinct subgroups with respect to outcome preferences. Differences across the preference profiles were marked: class 1 participants prioritized life expectancy over all other outcomes and appeared to be indifferent to most treatment burden outcomes, whereas people in class 2 prioritized preservation or improvement in lung function and in treatment burden reduction. Class 3 participants tended to have better overall health, which likely explains their tendency not to opt for an additional treatment in the experiment; however, interpretation of membership characteristics for class 3 should be cautious owing to its small size. There was a slight nominal trend for better overall health, HRQoL, and objective treatment burden in class 1 vs class 2; however, these were found to be poor predictors of class membership. Although this study was not designed or powered to address the question of population heterogeneity, our working hypothesis is that these differences in preference may be better explained by attitudinal, rather than clinical, characteristics.

This study may be prone to potential biases and limitations. As with all surveys there is a risk of response bias, for example, through answering strategically to influence policy.^36^ To mitigate this, the survey was designed in accordance with general good survey practice guidelines.^23^ Further, the broad agreement in relative importance of various aspects of treatment burden items of our findings with other studies suggests that any such bias is limited.^11^^,^^34^ Although the online questionnaire produced a good response rate of 37%, there may still be a nonresponse bias. However, there is a growing body of evidence suggesting little to no relationship between response rate and nonresponse bias.^37^ The use of a purposive, rather than a random, sampling approach poses a risk of selection bias, although in terms of clinical and demographic characteristics the sample is broadly representative of the population of adults with CF in the United Kingdom, with similar mean age, ppFEV_1_, and BMI.^38^ The co-incidental UK reimbursement decision for ETI during the study period may have introduced chronological bias, although no differences in preferences for the pre- and postlaunch segments of the sample were noted. DCEs focus on the stated preferences of participants in hypothetical scenarios; these may not truly reflect the choices that might be made in real life. Research into the external validity of DCEs is limited, although a meta-analysis suggested that well-designed experiments can predict choice reasonably well.^36^^,^^39^ Although we were unable to compare the stated preference results of this study with revealed preferences from the real world, it is our expectation that engagement of PwCF throughout the conceptualization, design, and piloting of the study has enhanced its external validity.

The study was designed with a sample sufficient to investigate main effects model, which assumes no interaction between attributes. Similarly, the study was not designed to assess preference heterogeneity in the primary analysis; the latent class results should therefore be interpreted as indicative.

As a single-center study, the generalizability of the findings presented to other settings here may be limited. However, the sample was similar to the overall UK CF population on the basis of key clinical and demographic parameters,^38^ and scores for health state utility (mean, 0.77), and treatment burden measured by the CFQ-R (mean, 54), were similar to those reported elsewhere for patients with mild disease (ppFEV_1_ ≥ 70).^32^ At the time this study was done, there was limited patient experience with ETI. Given the documented impact of ETI on treatment burden,^13^^,^^40^ the preferences presented in this study may be subject to change now that the eligible CF population is established on the treatment.

## Interpretation

The findings of this study add substantially to a very sparse literature on the preferences of PwCF, and suggest that, on average, they would trade benefits likely to be captured in conventional trials (ppFEV_1_) and economic evaluations (HRQoL, life expectancy) for benefits that might not be captured within current conventional evaluations (eg, reduced treatment burden and time). Further, our results indicate that PwCF are not a homogeneous group regarding the outcomes they prioritize: this has important equity implications when considering how patient values should inform decision-making, both on the ground at the clinic and at the health-system level. The study provides important evidence on the relative importance of outcomes from a patient perspective, which could be used alongside other scientific evidence in health technology appraisals to support decision-making for either the regulation or funding of CF treatments. Moreover, the comparative importance of treatment burden for patients suggests it should be considered as an important secondary outcome in CF when designing future prospective trials of novel therapies.

## References

[1] Scotet V., L’Hostis C., Ferec C. (2020). The changing epidemiology of cystic fibrosis: incidence, survival and impact of the CFTR gene discovery. Genes (Basel).

[2] Sawicki G.S., Sellers D.E., Robinson W.M. (2009). High treatment burden in adults with cystic fibrosis: challenges to disease self-management. J Cyst Fibros.

[3] Habib A.R., Manji J., Wilcox P.G., Javer A.R., Buxton J.A., Quon B.S. (2015). A systematic review of factors associated with health-related quality of life in adolescents and adults with cystic fibrosis. Ann Am Thorac Soc.

[4] Sawicki G.S., Ren C.L., Konstan M.W. (2013). Treatment complexity in cystic fibrosis: trends over time and associations with site-specific outcomes. J Cyst Fibros.

[5] Abbott J., Dodd M., Bilton D., Webb A.K. (1994). Treatment compliance in adults with cystic fibrosis. Thorax.

[6] White D., Stiller K., Haensel N. (2007). Adherence of adult cystic fibrosis patients with airway clearance and exercise regimens. J Cyst Fibros.

[7] Narayanan S., Mainz J.G., Gala S., Tabori H., Grossoehme D. (2017). Adherence to therapies in cystic fibrosis: a targeted literature review. Expert Rev Respir Med.

[8] Eakin M.N., Bilderback A., Boyle M.P., Mogayzel P.J., Riekert K.A. (2011). Longitudinal association between medication adherence and lung health in people with cystic fibrosis. J Cyst Fibros.

[9] Quittner A.L., Zhang J., Marynchenko M. (2014). Pulmonary medication adherence and health-care use in cystic fibrosis. Chest.

[10] May C., Montori V.M., Mair F.S. (2009). We need minimally disruptive medicine. BMJ.

[11] Rowbotham N.J., Smith S., Leighton P.A. (2018). The top 10 research priorities in cystic fibrosis developed by a partnership between people with CF and healthcare providers. Thorax.

[12] Hollin I.L., Donaldson S.H., Roman C. (2019). Beyond the expected: identifying broad research priorities of researchers and the cystic fibrosis community. J Cyst Fibros.

[13] Granger E., Davies G., Keogh R.H. (2022). Treatment patterns in people with cystic fibrosis: have they changed since the introduction of ivacaftor?. J Cystic Fibros.

[14] Gulland A. (2016). Cystic fibrosis drug is not cost effective, says NICE. BMJ.

[15] Huls S.P.I., Whichello C.L., van Exel J., Uyl-de Groot C.A., de Bekker-Grob E.W. (2019). What is next for patient preferences in health technology assessment? A systematic review of the challenges. Value Health.

[16] Chachoua L., Dabbous M., Francois C., Dussart C., Aballea S., Toumi M. (2020). Use of patient preference information in benefit-risk assessment, health technology assessment, and pricing and reimbursement decisions: a systematic literature review of attempts and initiatives. Front Med (Lausanne).

[17] Dirksen C.D., Utens C.M., Joore M.A. (2013). Integrating evidence on patient preferences in healthcare policy decisions: protocol of the patient-VIP study. Implement Sci.

[18] Carr S., Archangelidi O., Keogh K., Abbott J., Whitty J.A., Simmonds N.J. (2019).

[19] Amaya-Amaya M., Gerard K., Ryan M., Ryan M., Gerard K., Amaya-Amaya M. (2008). Using Discrete Choice Experiments to Value Health and Health Care.

[20] Lancaster K.J. (1966). A new approach to consumer theory. J Polit Econ.

[21] McFadden D. (2001). Economic choices. Am Econ Rev.

[22] Bridges J.F., Hauber A.B., Marshall D. (2011). Conjoint analysis applications in health—a checklist: a report of the ISPOR Good Research Practices for Conjoint Analysis Task Force. Value Health.

[23] Dillman D.A., Smyth J.D., Christian L.M. (2014).

[24] Middleton P.G., Mall M.A., Drevinek P. (2019). Elexacaftor-tezacaftor-ivacaftor for cystic fibrosis with a single Phe508del allele. N Engl J Med.

[25] Sawicki G.S., Rasouliyan L., McMullen A.H. (2011). Longitudinal assessment of health-related quality of life in an observational cohort of patients with cystic fibrosis. Pediatr Pulmonol.

[26] Altabee R., Carr S.B., Turner D. (2022). Exploring the nature of perceived treatment burden: a study to compare treatment burden measures in adults with cystic fibrosis [version 1; peer review: 1 approved]. NIHR Open Res.

[27] Hole A.R. (2007). A comparison of approaches to estimating confidence intervals for willingness to pay measures. Health Econ.

[28] Yoo H.I. (2019). https://ssrn.com/abstract=3484429.

[29] Hensher D.A., Rose J.M., Greene W.H. (2015).

[30] van Hout B., Janssen M.F., Feng Y.S. (2012). Interim scoring for the EQ-5D-5L: mapping the EQ-5D-5L to EQ-5D-3L value sets. Value Health.

[31] Shaikh N., Lee A., Charman S., Cosgriff R., Carr S. (2021).

[32] Acaster S., Pinder B., Mukuria C., Copans A. (2015). Mapping the EQ-5D index from the Cystic Fibrosis Questionnaire-Revised using multiple modelling approaches. Health Qual Life Outcomes.

[33] Lancsar E., Louviere J. (2006). Deleting “irrational” responses from discrete choice experiments: a case of investigating or imposing preferences?. Health Econ.

[34] Davies G., Rowbotham N.J., Smith S. (2020). Characterising burden of treatment in cystic fibrosis to identify priority areas for clinical trials. J Cyst Fibros.

[35] Ley D., Turck D. (2022). Digestive outcomes in cystic fibrosis. Best Pract Res Clin Gastroenterol.

[36] Quaife M., Terris-Prestholt F., Di Tanna G.L., Vickerman P. (2018). How well do discrete choice experiments predict health choices? A systematic review and meta-analysis of external validity. Eur J Health Econ.

[37] Hendra R., Hill A. (2019). Rethinking response rates: new evidence of little relationship between survey response rates and nonresponse bias. Eval Rev.

[38] Charman S., Lee A., Cosgriff R., McClenaghan E., Carr S. (2020).

[39] Lancsar E., Swait J. (2014). Reconceptualising the external validity of discrete choice experiments. Pharmacoeconomics.

[40] Martin C., Reynaud-Gaubert M., Hamidfar R. (2022). Sustained effectiveness of elexacaftor-tezacaftor-ivacaftor in lung transplant candidates with cystic fibrosis. J Cyst Fibros.

