# Supplementary material for: Treatment Preference Among People With Cystic Fibrosis: The Importance of Reducing Treatment Burden
Source: Chest. 2022 Jul 19;162(6):1241–54. doi: 10.1016/j.chest.2022.07.008 (PMC9773229; doi:10.1016/j.chest.2022.07.008)
Supplement: Supplementary File [file mmc1.docx]

# SUPPLEMENTARY FILE

## e-Appendix 1:

**Experimental Design**

Efficient designs facilitate model estimation with smaller sample sizes than alternative experimental design methods.^1^ The design assumed that the primary model would be a multinomial logit model (MNL), estimating main effects with dummy coded variables. Initially, the prior parameters for the design were set at near-0 positive values for all parameters except the lung function attribute level that indicated a 5% decrease in ppFEV_1_, which was assumed to be negative. After 35 participants had completed the survey, a multinomial logit (MNL) model was estimated, and its coefficients used to provide Bayesian priors for an updated experimental design. The generated design comprised 36 choice pairs, grouped into 3 blocks, with each participant randomly assigned to one of the three blocks of 12 choice scenarios.

## e-Appendix 2:

**Treatment Complexity Score**

The treatment complexity score (TCS) was developed by Sawicki *et al.^2^* as a means to quantify the effort involved in using CF medications, based on their frequency, administration time, and method. Each treatment is assigned a complexity of 1 (low complexity), 2, or 3 (high complexity). Scores for each treatment are summed to give an overall TCS for each participant. High TCS score suggests high treatment complexity. TCS scoring in this study was based on the original, although with some modification,^3^ to add treatments that were not in the original (Table e1)

1. Bliemer MCJ, Rose JM. Efficiency and Sample Size Requirements for Stated Choice Studies. In:2005.

2. Sawicki GS, Ren CL, Konstan MW, et al. Treatment complexity in cystic fibrosis: trends over time and associations with site-specific outcomes. *J Cyst Fibros.* 2013;12(5):461-467.

3. Altabee R, Carr SB, Turner D, et al. Exploring the nature of perceived treatment burden: a study to compare treatment burden measures in adults with cystic fibrosis In. Manuscript in preparation2021.

## e-Table 1

**The modified version of Sawicki *et al*.’s treatment complexity score table**

| TCS Score = 1 point | TCS Score = 2 points | TCS Score = 3 points |
| --- | --- | --- |
| Acid blockers | Antibiotics (nebulized OD) | Antifungals (inhaled) * |
| Analgesics | DNase (OD / OR) | Antibiotics (nebulized (BD/TDS) |
| Angiotensin receptor agonists * | Hypertonic saline (OD) | DNase (BD) * |
| Antibiotics (inhaled DPI) * | Pancreatic enzymes | Hypertonic saline (BD) * |
| Anticoagulants * | CFTR modulator * | Mannitol (DPI) |
| Antidepressants |  | Insulin |
| Antiemetics * |  | Colistin (nebulized) * |
| Antiepileptic * |  | Oxygen |
| Antifungals (oral) * |  | Airway clearance |
| Antihistamines * |  | Noninvasive ventilation * |
| Anti-inflammatories * |  |  |
| Antiviral * |  |  |
| Beta blocker * |  |  |
| Bisphosphonates * |  |  |
| Bronchodilators (inhaled) |  |  |
| Bronchodilators (oral) |  |  |
| Chronic oral antibiotics |  |  |
| Corticosteroids (inhaled) |  |  |
| Corticosteroids (inhaled) + LABA |  |  |
| Corticosteroids (oral) |  |  |
| Diuretics |  |  |
| Immunosuppressants (oral) * |  |  |
| Tranexamic acid 1 gm (TDS, PRN) * |  |  |
| Metformin * |  |  |
| Migraine prophylaxis * |  |  |
| Minerals (oral) |  |  |
| Nasal rinse/ spray * |  |  |
| Prophylactic antibiotics (oral) |  |  |
| Ropinirole * |  |  |
| Statin * |  |  |
| Tamoxifen * |  |  |
| Vitamins (oral) |  |  |
| Gastrointestinal medicines * |  |  |

* The newly added treatments to Sawicki et al.’s original version – none of the assigned treatments from the original version were moved to different categories or removed from the scale.

Abbreviations: TCS = treatment complexity score, DPI = dry powder inhaler, LABA = long-acting beta agonist, TDS = three times a day, PRN = as required, OD = once a day, OR = other regimen, BD = twice a day.

## e-Table 2

**Comparison of characteristics of participants with those of Royal Brompton Hospital Adult CF Centre**

|  | Survey Sample  N=101 | RBH Adult CF Centre  N=550 |
| --- | --- | --- |
| Clinical measures |  |  |
| ppFEV1  Unadjusted mean  Median | 69.5  69.2 | 67.0  68 |
| BMI  Unadjusted mean  Median | 22.8  22.5 | 23.0  22.5 |
| Treatment characteristics |  |  |
| Prescribed DNase | 91% | 90.9% |
| Prescribed hypertonic saline / mannitol | 60% | 56.0% |

## e-Table 3

**MNL Model 2 with continuous variables for lung function & life expectancy**

| Attribute | Parameter (level) | Marginal effect | 95% CI |
| --- | --- | --- | --- |
| Opt-out | - | 0.44 | -0.21, 1.09 |
| Lung function | per 1% change in lung function | 0.08*** | 0.06, 0.11 |
| Need for IV antibiotics | no change | referent |  |
|  | half the number of IV courses | 0.2** | 0.04, 0.36 |
| Abdominal symptoms | no change | referent |  |
|  | improvement in symptoms | 0.35** | 0.12, 0.59 |
|  | improvement in symptoms and a reduction in pancreatic enzymes | 0.44*** | 0.25, 0.63 |
| Life expectancy | per 1 year increase in life expectancy | 0.16*** | 0.13, 0.20 |
| Overall quality of life | no change | referent |  |
|  | good improvement (+10%) | 0.29** | 0.10, 0.48 |
|  | excellent improvement (+20%) | 0.69*** | 0.47, 0.91 |
| Use of inhaled medicines | no change | referent |  |
|  | a modest reduction in time spent (-25%) | 0.16* | 0.00, 0.32 |
|  | a large reduction in time spent (-50%) | 0.37*** | 0.20, 0.54 |
| Physio/ ACT | no change | referent |  |
|  | time spent on physio is halved | 0.23*** | 0.11, 0.35 |
|  | able to fully stop physio | 0.51*** | 0.27, 0.75 |
| Model statistics | No. Observations: 1236; McFadden’s R^2^: 0.31; LL: -933; AIC: 1892; BIC: 1973 | | |
| LL: log likelihood; AIC: Akaike information criteria; BIC: Bayesian information criteria  * *P* <0.1 ** p<0.05 *** p<0.001 | | | |

## e-Table 4

**Willingness to accept a reduction in ppFEV1 or additional life expectancy against other treatment outcomes, for those prescribed or not prescribed CFTR modulators**

|  | Acceptable reduction in ppFEV1 (95% CI) | | Acceptable reduction additional life expectancy^a^ (95% CI) | |
| --- | --- | --- | --- | --- |
| Attribute | Prescribed CFTR  N=66 | Not prescribed CFTR  N=35 | Prescribed CFTR  N=66 | Not prescribed CFTR  N=35 |
| Excellent improvement (+20%) in QoL | 6.5 (3.7 to 9.2) | 11.4 (6.1 to 16.6) | 3.4 (2.1 to 4.6) | 5.8 (3.4 to 8.3) |
| Able to fully stop physio | 6.3 (2.8 to 9.8) | 5.9 (2.2 to 9.6) | 3.3 (1.5 to 5.1) | 3.1 (1.0 to 5.1) |
| Abdominal symptoms improved and enzymes reduced | 3.9 (1.4 to 6.4) | 8.2 (4.4 to 12.0) | 2.0 (0.6 to 3.4) | 4.2 (2.4 to 6.1) |
| A large reduction in time spent (-50%) on inhaled medicines | 4.8 (2.3 to 7.3) | 3.5 (0.7 to 6.2) | 2.5 (1.1 to 3.8) | 1.8 (0.4 to 3.2) |
| Abdominal symptoms improved | 2.2 (1.2 to 5.6) | 7.8 (3.4 to 12.1) | 1.1 (0.7 to 2.9) | 4.0 (1.7 to 6.3) |
| Good improvement (+10%) in QoL | 2.6 (0.3 to 5.5) | 4.6 (0.5 to 8.8) | 1.3 (0.1 to 2.8) | 2.4 (0.4 to 4.4) |
| Time spent on physio is halved | 2.2 (0.4 to 4.0) | 3.2 (0.2 to 6.2) | 1.1 (0.2 to 2.0) | 1.6 (0.3 to 3.0) |
| IV days halved | 2.1 (0.2 to 4.4) | 2.9 (0.5 to 5.3) | 1.1 (0.2 to 2.3) | 1.5 (0.1 to 2.9) |
| Per year increase in life expectancy | 1.9 (1.4 to 2.5) | 1.9 (1.3 to 2.6) | - | - |
| A modest reduction in time spent (-25%) on inhaled medicines | 1.2 (1.3 to 3.8) | 2.7 (0.1 to 5.5) | 0.6 (-0.7 to 2.0) | 1.4 (0.1 to 2.9) |
| Per 1% increase in predicted FEV1 | - | - | 0.5 (0.4 to 0.7) | 0.5 (0.4 to 0.7) |
| ^a^Additional life expectancy should be interpreted as the additional life expectancy conferred by the hypothetical treatments presented in the DCE, beyond existing life expectancy | | | | |

## e-Table 5

**Comparison of characteristics of participants prescribed and not prescribed a CFTR modulator**

|  | Prescribed CFTR  N=66 | Not prescribed CFTR  N=35 |
| --- | --- | --- |
| Demographics |  |  |
| Age (years) | 36 | 36 |
| Gender (% female) | 45 | 63 |
| Clinical measures |  |  |
| ppFEV1 | 68 | 72 |
| absFEV1 (l) | 2.5 | 2.4 |
| ppFVC | 84 | 88 |
| absFVC (l) | 3.7 | 3.5 |
| Diagnosis of GERD (%) | 41 | 43 |
| Diagnosis of CFRD (%) | 30 | 26 |
| BMI | 23 | 23 |
| Treatment characteristics |  |  |
| Treatment complexity score | 24 | 19** |
| Total treatment time (mins/day) | 104 | 71** |
| Physiotherapy time (mins/day) | 41 | 29* |
| Inhaled medicines time (mins/day) | 47 | 37 |
| No. chronic treatments | 14 | 11** |
| Received IV antibiotics in last year (%) | 67 | 60 |
| Number of IV antibiotic courses in last year | 3 | 2 |
| HRQoL and treatment burden measures |  |  |
| EQ-5D Index score | 0.76 | 0.79 |
| EQ-5D VAS score | 75 | 75 |
| CFQ-R treatment burden domain score | 51 | 57 |
| CFQoL treatment burden domain score | 62 | 68 |
| MTBQ index score (reversed) | 82 | 80 |
| * *P* <0.1 ** *P* <0.05 | | |

## e-Figure 1

**MNL Model 1 results stratified by those prescribed or not prescribed CFTR modulators**

Values are mean marginal effect, ± 95% CI. The marginal effect for no change (reference level) is represented by the dashed horizontal line. Abd. sympt, abdominal symptoms; LE, life expectancy (years); QoL, overall quality of life; Inh. meds, inhaled medications; Physio, physiotherapy; impr, symptoms improved; impr. enzymes reduced, symptoms reduced and pancreatic enzymes reduced.

##

## e-Figure 2

**MNL Model 1 results stratified by those completing before and after licensing of elexacaftor-tezacaftor-ivacaftor**

Values are mean marginal effect, ± 95% CI. The marginal effect for no change (reference level) is represented by the dashed horizontal line. Abd. sympt, abdominal symptoms; LE, life expectancy (years); QoL, overall quality of life; Inh. meds, inhaled medications; Physio, physiotherapy; impr, symptoms improved; impr. enzymes reduced, symptoms reduced and pancreatic enzymes reduced.

##
